# Supplementary material for: The interactome of intact mitochondria by cross-linking mass spectrometry provides evidence for coexisting respiratory supercomplexes
Source: Mol Cell Proteomics. 2017 Dec 8;17(2):216–32. doi: 10.1074/mcp.RA117.000470 (PMC5795388; doi:10.1074/mcp.RA117.000470)
Supplement: Supplemental Data [file supp_RA117.000470_133922_0_supp_23978_fzffwf.zip › spectra_annotation/mito_DR_spectra_annotation/48-1-9-1-8-1.pdf]

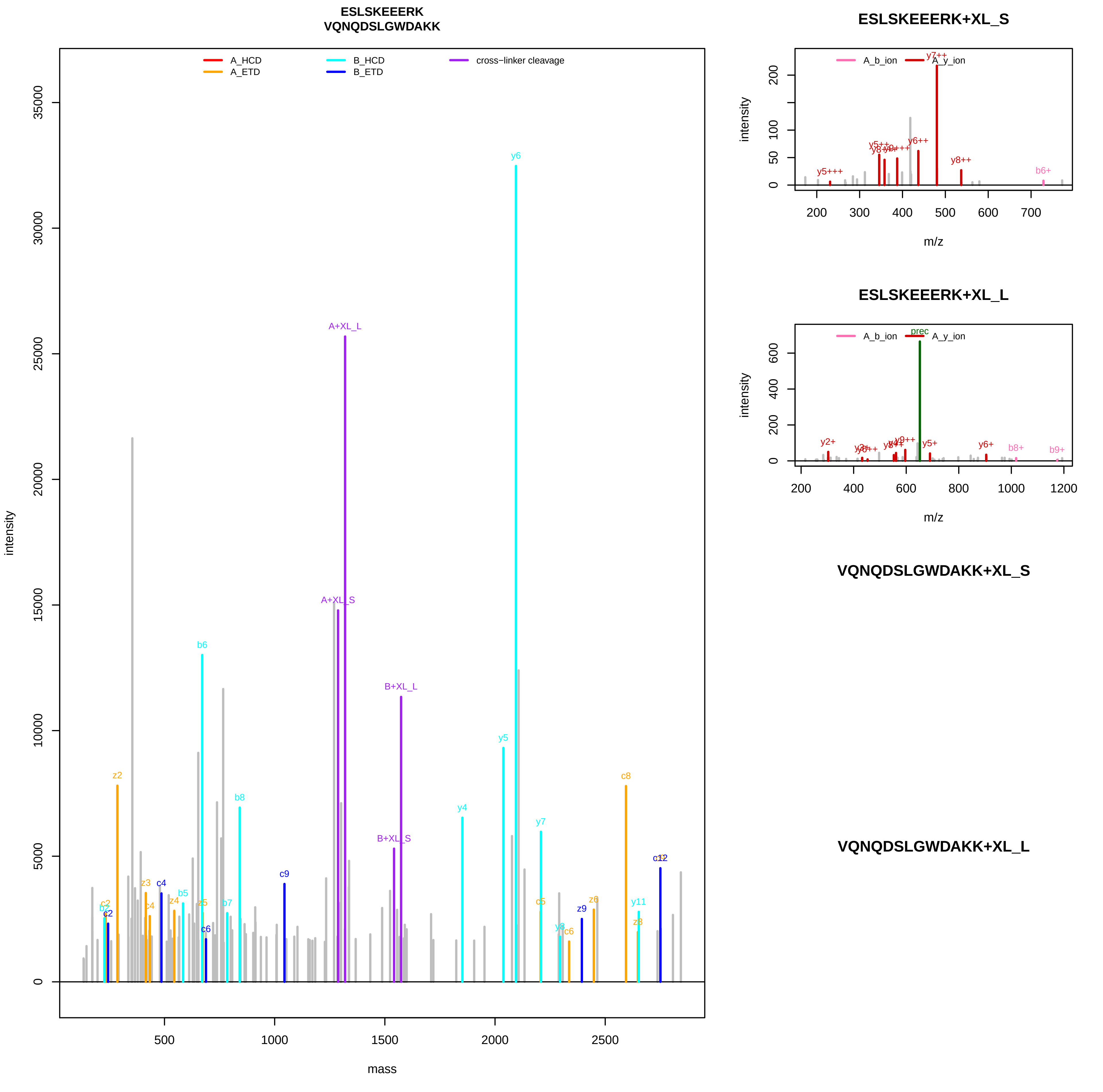

ESLSKEEERK
VQNQDSLGWDAKK
A_HCD
A_ETD
B_HCD
B_ETD
cross−linker cleavage
intensity
mass
A+XL_L
A+XL_S
B+XL_L
B+XL_S
y6
y5
y4
y7
b6
b8
z2
c8
c9
c12
y11
ESLSKEEERK+XL_S
A_b_ion
A_y_ion
y7++
y6++
y8++
y5+++
b6+
m/z
ESLSKEEERK+XL_L
prec
y2+
y5+
y6+
b8+
b9+
VQNQDSLGWDAKK+XL_S
VQNQDSLGWDAKK+XL_L
